# *In vivo* Expansion of Regulatory T cells via an Engineered IL-2 Mutein to Suppress Autoimmune Glomerulonephritis

**DOI:** 10.7150/thno.126588

**Published:** 2026-02-18

**Authors:** Huang Kuang, Cai-Xia Lin, Jing Huang, Wen-Xuan Li, Yu-Ge Zhu, Nan Jiang, Zhong Li, Nan Li, Ping Li, Xiao-Yu Jia, Zhao Cui, Ming-Hui Zhao

**Affiliations:** 1 Renal Division, Peking University First Hospital, Beijing 100034, China.; 2 Institute of Nephrology, Peking University, Beijing 100034, China.; 3 Key Laboratory of Renal Disease, Ministry of Health of China, Beijing 100034, China.; 4 Key Laboratory of CKD Prevention and Treatment, Ministry of Education of China, Beijing 100034, China.; 5 Key Laboratory of Carcinogenesis and Translational Research (Ministry of Education/Beijing), Department of Thoracic Surgery II, Peking University Cancer Hospital & Institute, Beijing 100142, China.; 6 Staidson (Beijing) Biopharmaceuticals Co., Ltd, Beijing 100176, China.

**Keywords:** regulatory T cells, interleukin-2 mutein, autoimmunity, autoimmune glomerulonephritis, immunotherapy

## Abstract

**Rationale:**

Regulatory T (Treg) cells suppress autoimmunity and restrain inflammatory responses, showing promising potential in autoimmune glomerulonephritis (GN) therapy with minimizing nonspecific immunosuppression. Although low-dose interleukin-2 (IL-2) has been shown to promote Treg expansion, its clinical utility is constrained by its short half-life and concurrent effector T cell activation.

**Methods:**

An IL-2 mutein STS718 was engineered by introducing point mutations and fusing it to a human IgG1 Fc domain. The molecular characteristics of STS718, including its affinity, selectivity, and half-life were evaluated. *In vivo* expansion of Treg cells by STS718 was assessed in mice and cynomolgus monkeys. Experimental autoimmune GN models, including crescentic GN and membrane GN, were established to test the therapeutic potential of STS718. The ability of STS718 to induce human functional Treg cells was confirmed using human naïve CD4^+^ T cells from donors and peripheral blood mononuclear cells (PBMCs) from autoimmune GN patients.

**Results:**

STS718 exhibited a lower affinity for IL-2 receptor (IL-2Rβγ) and comparable affinity for IL-2Rαβγ compared with wild-type IL-2-Fc of human, rat, and mouse, as well as a prolonged half-life. STS718 expanded Treg cells in mice and cynomolgus monkeys in a manner that was dependent on either time or dose, without significantly affecting the effector T cell activation. Proof-of-concept experiments confirmed that sustained Treg expansion mediated by STS718 effectively suppressed the progression of autoimmune GN models, exhibiting superior efficacy compared to wild-type IL-2-Fc. In addition, the STS718 was capable of inducing the expansion of human functional Treg cells from either naïve CD4^+^ T cells of healthy donors or PBMCs from autoimmune GN patients.

**Conclusions:**

Collectively, these findings suggest that engineered IL-2 mutein which selectively expands Treg cells *in vivo* holds significant promise as an alternative immunotherapeutic strategy for controlling autoimmune GN while reducing nonspecific immunosuppression.

## Introduction

Autoimmune glomerulonephritis (GN) represents a major subset of all GN, such as membranous GN, crescentic GN, and IgA nephropathy. It is characterized by an adaptive immune response that is directed against multiple different autoantigens 1. Across these disorders, the central immunopathogenesis involves the breakdown of immune tolerance and overactivation of autoreactive T cells or B cells, leading to inflammation attack of the kidneys 2-4. Despite the evolving understanding of the immunopathogenesis of autoimmune GN, nonspecific immunosuppression—such as glucocorticoids and cyclophosphamide which are linked to considerable side effects and imprecise immunomodulation—remain the most common treatment regimen of affected patients. Suboptimal treatment often leads autoimmune GN patients to progress into end-stage kidney disease and require lifelong dialysis. Alternative therapies that induce tolerance restoration and selectively target adaptive immunity remain scarce but vital.

Regulatory T (Treg) cells, characterized by expression of the transcription factor forkhead box protein 3 (Foxp3), play a critical role in maintaining immunological self-tolerance and modulating antigen-specific immune responses 5, 6. Their unique properties make Treg-based therapies attractive alternatives for minimizing nonspecific immunosuppression, or even serve as frontline immunotherapy in autoimmune GN. Indeed, the therapeutic capacity of Treg cells has been validated across multiple preclinical autoimmune models, including graftversushost disease 7-9, autoimmune diabetes 10-13, systemic lupus erythematosus 14, 15, and multiple sclerosis 16, 17. Several strategies have been developed to induce and amplify Treg cells, among which interleukin-2 (IL-2)-based immunotherapy has been a primary focus in clinical development for the treatment of autoimmune disorders 18. This immunotherapy is based on the idea that IL-2 is crucial for the development, homeostasis, and function of Treg cells. IL-2 signaling is transduced through its receptor (IL-2R), which can exist in either heterodimeric or heterotrimeric form 19. The dimeric IL-2R, consisting of the IL-2Rβ chain (CD122) and the γ chain (CD132) has low affinity for IL-2, while the trimeric IL-2R, which additionally includes the IL-2Rα chain (CD25), has high affinity for IL-2 20. Physiologically, high levels of IL-2Rβγ expression have been observed in CD8^+^ T cells (both naïve and memory subsets), memory CD4^+^ T cells, and CD56^low^ NK cells, making these subsets sensitive to exogenously administrated IL-2 21. In contrast, Treg cells constitutively express trimeric IL-2Rαβγ, and IL-2 signaling could function under physiological IL-2 levels. A major challenge, therefore, is how to adjust the IL-2 dosing to preferentially expand Treg cells rather than effector cells. Moreover, short serum half-life of IL-2 is another obstacle for restoring self-tolerance, since sustained benefits require frequent administration. These limitations have prompted the advancement of engineered IL-2 products to enhance Treg selectivity and favorable pharmacodynamics.

In the current study, we designed an engineered IL-2 mutein conjugated with the human IgG Fc domain to reduce its affinity for IL-2Rβγ and prolong its half-life. Our findings suggest that this IL-2 mutein (STS718) is capable of selectively expanding Treg cells *in vivo* and suppressing severe inflammation of experimental autoimmune GN, providing important proof-of-concept insights for IL-2-based immunotherapy in treating autoimmune GN patients.

## Materials and Methods

### Design and selection of IL-2 mutein

DNA sequences encoding wild-type IL-2 protein and its receptor subunits (IL-2Rα, IL-2Rβ, and IL-2Rγ) were synthesized (GenScript) with a His-tag and sub-cloned into the pcDNA3.1 plasmid (Thermo Fisher Scientific). Plasmid was transfected into HEK-293F cells and supernatant was collected to purify these proteins using Ni-NTA chromatography.

The IL-2Rβγ dimer and IL-2Rαβγ trimer were constructed by Knob-into-Hole technology. Briefly, for IL-2Rβγ dimer, extracellular domains of IL-2Rβ and IL-2Rγ were fused with N-terminal of Fc hole (SEQ ID NO: 109) and Fc knob (SEQ ID NO: 108) respectively. Plasmids of IL-2Rβ-Fc hole and IL-2Rγ-Fc knob were then co-transfected into HEK-293F cells. IL-2Rαβγ trimer was constructed by IL-2Rαγ-Fc knob and IL-2Rβ-Fc hole. Both dimer and trimer were purified from cell culture supernatant using protein A affinity chromatography.

A library of designed IL-2 muteins was constructed into yeast surface display (YSD) and screened with the following strategies: (i) decreased affinity for biotinylated IL-2βγ; (ii) essentially unchanged or not significantly diminished affinity for biotinylated IL-2Rα. The binding was detected with Streptavidin-PE and V5-tag antibodies by fluorescence-activated cell sorting (FACS). Screened clone was further sequenced and analyzed. The screened ideal IL-2 mutein, together with wild-type IL-2 were ligated to human IgG1 Fc (mutations in L234A and L235A abolished effector functions by eliminating binding to C1q and Fcγ receptor while preserving the normal interaction with FcRn 22) via a linker peptide (GGGGS), respectively. Gene sequences of IL-2 mutein-Fc, designated as STS718, and wild-type IL-2-Fc were synthesized, sub-cloned into pcDNA3.1 plasmid, and then transfected into HEK293F cells. STS718 and wild-type IL-2-Fc were purified from cell culture supernatant by protein A affinity chromatography, followed by size-exclusion chromatography on a Superdex 200 column. Endotoxin of purified STS718 used *in vivo* experiments contained was ≤ 1 units (EU)/mg.

### *In vitro* binding affinity analysis and biological activity analysis of IL-2 mutein

Binding affinities of STS718 and wild-type IL-2-Fc to human, rat and mouse IL-2βγ and IL-2Rαβγ were measured via surface plasmon resonance (SPR) on a Biacore T200 instrument (GE Healthcare). IL-2Rβγ-Fc or IL-2Rαβγ-Fc was captured on Protein A-immobilized chip, and a series of concentrations of STS718 or wild-type IL-2-Fc were then flowed over the chip surface. Data were recorded and binding affinities (K_D_) were determined via the Biacore T200 Evaluation software.

HEK-Blue™ IL-2 reporter cells (hkb-il2; Invitrogen) expressing IL-2Rαβγ or HEK-Blue™ CD122/CD132 reporter cells (hkb-il2bg; Invitrogen) expressing IL-2Rβγ were used to assess the biological activity of STS718 or wild-type IL-2-Fc. Phosphorylated signal transducer and activator of transcription (pSTAT5) levels were detected by the secreted SEAP (secreted embryonic alkaline phosphatase) signal in reporter cells. SEAP signal (OD value) was read at 620 nm and EC_50_ (median effective concentration) was calculated. Detailed information for antibodies and relevant reagents was provided in **Table S1**. In the current study, Treg cells were defined as CD4^+^CD25^+^Foxp3^+^ cells.

Human peripheral blood mononuclear cells (PBMCs), sourced from Milecell Bio (Shanghai, China), were used to assess the bioactivity of STS718 and wild-type IL-2-Fc via the detection of pSTAT5 levels. Briefly, PBMCs were cultured with complete RPMI 1640 medium with 2% FBS in a 96-well plate (5.0×10^5^ cells/well) and exposed to a concentration gradient of wild-type IL-2-Fc and STS718 for 30 min. Following stimulation, cells were then harvested and stained using a defined panel of antibodies for the analysis of CD8^+^ T cells and NK cells (CD3-BV421, CD8-PE, CD56-AF488, and pSTAT5-AF647), and a different panel for the analysis of Treg cells (CD4-BV421, CD25-AF647, Foxp3-AF488, and pSTAT5-PE). pSTAT5 MFI (mean fluorescence intensity) was quantified through flow cytometry and analyzed with FlowJo software v10.8, with corresponding EC_50_ values were calculated.

### Pharmacokinetics Study of IL-2 mutein

C57BL/6J mice and Sprague-Dawley (SD) rats were obtained from the Charles River Laboratories. Mice and rats were administered a single intravenous dose of STS718 (1 mg/kg). Blood was sampled from all animals at 0, 1, 5, 24, 72, and 168 h following administrations. Pharmacokinetic parameters were determined from plasma by non-compartmental analysis using Phoenix® WinNonlin® (Certara USA).

### *In vivo* Treg expansion of IL-2 mutein in mice and nonhuman primates

The bioactivity of STS718 to expand Treg cells* in vivo* was evaluated in C57BL/6J mice (Charles River Laboratories) and cynomolgus monkeys (Beijing Prima Biotech). C57BL/6J mice were given three doses of STS718 (0.1, 0.25, and 0.5 mg/kg) at days 0 and 5, or PBS via intravenous injection. Blood was collected from pre-treatment mice (day -2) as well as on days 3 and 8 following administration. PBMCs were assessed via flow cytometry for surface and intracellular markers, including CD4-FITC, CD25-APC, Foxp3-PE, IL4-APC, IFNγ-BV421, and IL17A-PE. Cynomolgus monkeys were administered 0.3 mg/kg STS718 subcutaneously on day 0, followed by a dose of 1.0 mg/kg on day 14. Blood was sampled on days 0, 4, 7, 10, 14, 18, 21, and 24. PBMCs were assessed for expression of CD4-Pacific Blue™, CD25-AF647, Foxp3-AF488, and CD8-BV605™ by flow cytometry.

### Experimental autoimmune glomerulonephritis

Experimental crescentic glomerulonephritis (CrN) 23, 24 and passive Heymann nephritis (PHN) 25, 26 were established as previously described. Animal experiments were performed in compliance with the guidelines of the Institutional Animal Care and Use Committee of Peking University First Hospital (No. J2023055). Briefly, Wistar-Kyoto (WKY) rats and SD rats were obtained from Charles River Laboratories. For CrN induction, WKY rats were immunized with 0.4 µg/g of α3(IV)NC1_127-148_ (non-collagenous domain 1 of α3 chain of type IV collagen) peptide (Scilight Biotechnology) emulsified with complete Freund's adjuvant (CFA, Sigma-Aldrich) in both hind footpads. For PHN induction, SD rats were administered a single intravenous dose of sheep anti-rat Fx1A serum at a dose of 0.4 mL/100 g (PTX-002S, Probetex). Rats were randomized to different groups after immunization. For treatments, 0.3 mg/kg STS718 or wild-type IL-2-Fc or vehicle PBS was administered twice a week in WKY rats for CrN model or once a week in SD rats for PHN model from the day 0. To evaluate the efficacy of STS718 in established disease, PHN rats received 0.3 mg/kg STS718 from week 3 onwards, by which time severe proteinuria had developed. Experiments were ended at week 6. For disease assessment, 24-h urine and blood were sampled weekly. Clinical manifestation and kidney histopathology for both experimental autoimmune GN models were investigated as described previously 26, 27. Detailed methods for animal experiments were provided in **Supplemental Methods**.

### *In vitro* expansion of human Treg cells from naïve CD4^+^ T cells and PBMCs of healthy donors

Human naïve CD4^+^ T cells were purified from healthy donors with EasySep™ Human Naïve CD4^+^ T Cell Isolation Kit II (17555; StemCell). Naïve CD4^+^ T cells were activated using ImmunoCult™ Human CD3/CD28/CD2 T Cell Activator (10970; StemCell) in RPMI 1640 medium (Gibco) containing 5% FBS, 1% penicillin/streptomycin, and 50 µM β-mercaptoethanol), and cultured with 5 ng/mL transforming growth factor β1 (TGF-β1) (100-21; PeproTech) for 6 days. Cells were treated with 100 ng/mL STS718. Cells were then rested for one day in the absence of T cell activators and TGF-β1. On day 7, cells were harvested and analyzed via flow cytometry for expression of CD4-FITC, CD127-Percp/cy5.5, CD25-PE-Cy-7, and Foxp3-PE (panel 1), as well as CD4-FITC, PD1-PE-Cy7, CTLA4-APC, Foxp3-PE (panel 2). For the settings of isolated PBMCs from healthy donors, cells were stimulated with T Cell Activator and 5 ng/mL TGF-β1, together with 100 ng/mL STS718 for 4 days. After resting, cells were harvested and analyzed using the same flow cytometry panels as for human naïve T cells.

### *In vitro* expansion of Treg cells from PBMCs of autoimmune glomerulonephritis patients

Fresh PBMCs were isolated from autoimmune GN patients including membranous GN and antiglomerular basement membrane (anti-GBM) disease, were isolated with Lymphoprep in SepMate tubes (85450; StemCell). Isolated PBMCs were activated using the T Cell Activator and 5 ng/mL TGF-β1 for 4 days. Cells were incubated with 10 ng/mL recombinant human IL-2 (rhIL-2) and 100 ng/mL STS718. After resting and harvesting, the proportion of CD4^+^CD25^+^Foxp3^+^ Treg cells was analyzed, as described above. All patients were diagnosed at our hospital and gave written informed consent. Demographic information of these patients was described in **Table S2** and **Table S3**.

### Statistical Analysis

Statistical analysis was performed using GraphPad Prism 8.0 software. For titration curves of *in vitro* biological activity evaluation of STS718, EC_50_ values were derived by nonlinear fitting using a four-parameter variable-slope mode after transforming the concentration values to logarithms. Three technical replicates were set up and averaged for all *in vitro* experiments. Comprehensive statistical details were included in the figure legends. Statistical significance was defined as *P* value < 0.05, with the following notation: ns, not significant, *P < 0.05, **P < 0.01, ***P < 0.001, and ****P < 0.0001.

## Results

### Design of an IL-2 mutein STS718

**Figure 1:**
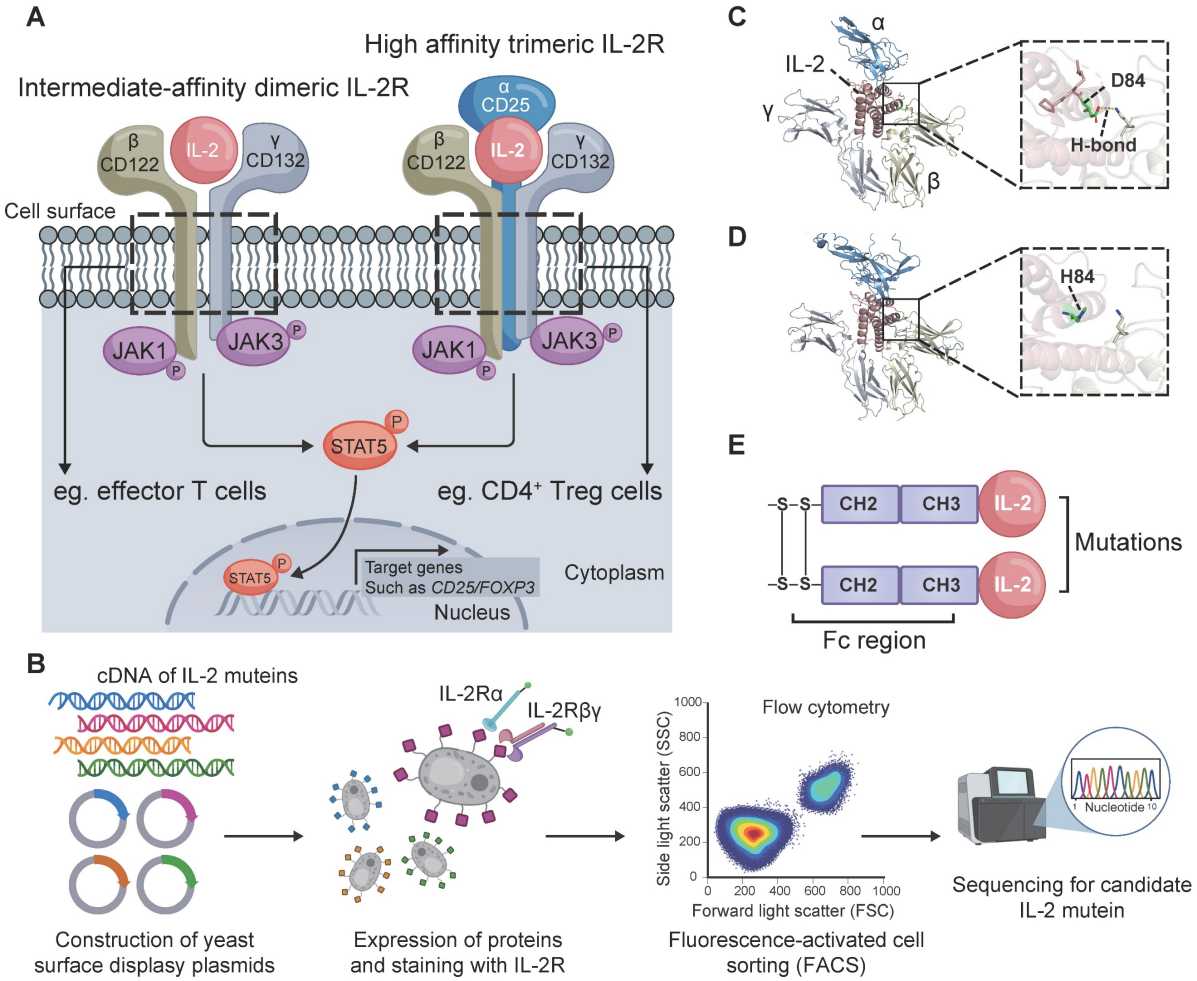
** Core IL-2 signaling and the design and structure of STS718.** (**A**) The intermediate-affinity dimeric IL-2 receptor (IL-2R), IL-2Rβγ, is constitutively expressed on effector cells, such as CD8^+^ T cells. Regulatory T (Treg) cells constitutively express the high-affinity trimeric IL-2R, IL-2Rαβγ. IL-2 induces the transcriptional expression of target genes such as CD25 and forkhead box protein 3 (Foxp3) through several pathways, with the Janus activating kinase 3 (JAK3) / signal transducer and activator of transcription 5 (STAT5) pathway being the primary one. (**B**) Schematic representation of STS718 design. Created with Biorender.com. (**C**) In wild-type human IL-2, the carboxyl oxygen atom of D84 formed a hydrogen bond (H-bond) with IL-2Rβ. (**D**) After point mutations, the 81RPRDL85 in wide-type IL-2 was substituted by RHL, and H84 was unable to form the H-bond between this mutein and IL-2Rβ. Structure model was constructed using AlphaFold 3 and images were generated with PyMOL. (**E**) Schematic of the structure of STS718.

Treg cells constitutively express the high-affinity trimeric IL-2Rαβγ, while effector immune cells such as CD8^+^ T cells predominantly express intermediate-affinity dimeric IL-2Rβγ (**Figure 1A**). We thus sought to engineer an IL-2 mutein that exhibits decreased affinity for IL-2Rβγ while maintaining affinity for IL-2Rα. A library of candidate muteins was constructed by YSD technology and then screened using FACS (**Figure 1B**). We finally obtained an enriched monoclonal yeast clone with reduced binding to IL-2Rβγ and sustained binding to IL-2Rα. The selected monoclonal clone was sequenced to analyze the mutant information. We found that this mutein undergone a mutation in the amino acids at position 81-85 relative to the human wild-type IL-2 counterpart, with 81RPRDL85 replaced by RHL. In wild-type human IL-2, the carboxyl oxygen atom of the aspartic acid at position 84 (D84) formed a 2.9 Å hydrogen bond with IL-2Rβ (**Figure 1C**). The substitution of D84 with histidine (H84) abolished the hydrogen bond interaction between this mutein and IL-2Rβ, thus potentially resulting in decreased binding affinity (**Figure 1D**). Additionally, a C125A mutation was incorporated into this mutein in order to promote effective protein folding and expression 28. Short *in vivo* half-life was another drawback for IL-2 immunotherapy 29. To address this, the screened IL-2 mutein was fused to human IgG1 Fc domain that contained L234A and L235A mutations to abolish its effector function 22. The IL-2-Fc mutein, termed STS718 (**Figure 1E**), was then expressed in human HEK-293F cells, and purified for further analysis.

### STS718 has reduced binding to IL-2Rβγ, preferential bioactivity on Treg cells, and a prolonged half-life

**Figure 2:**
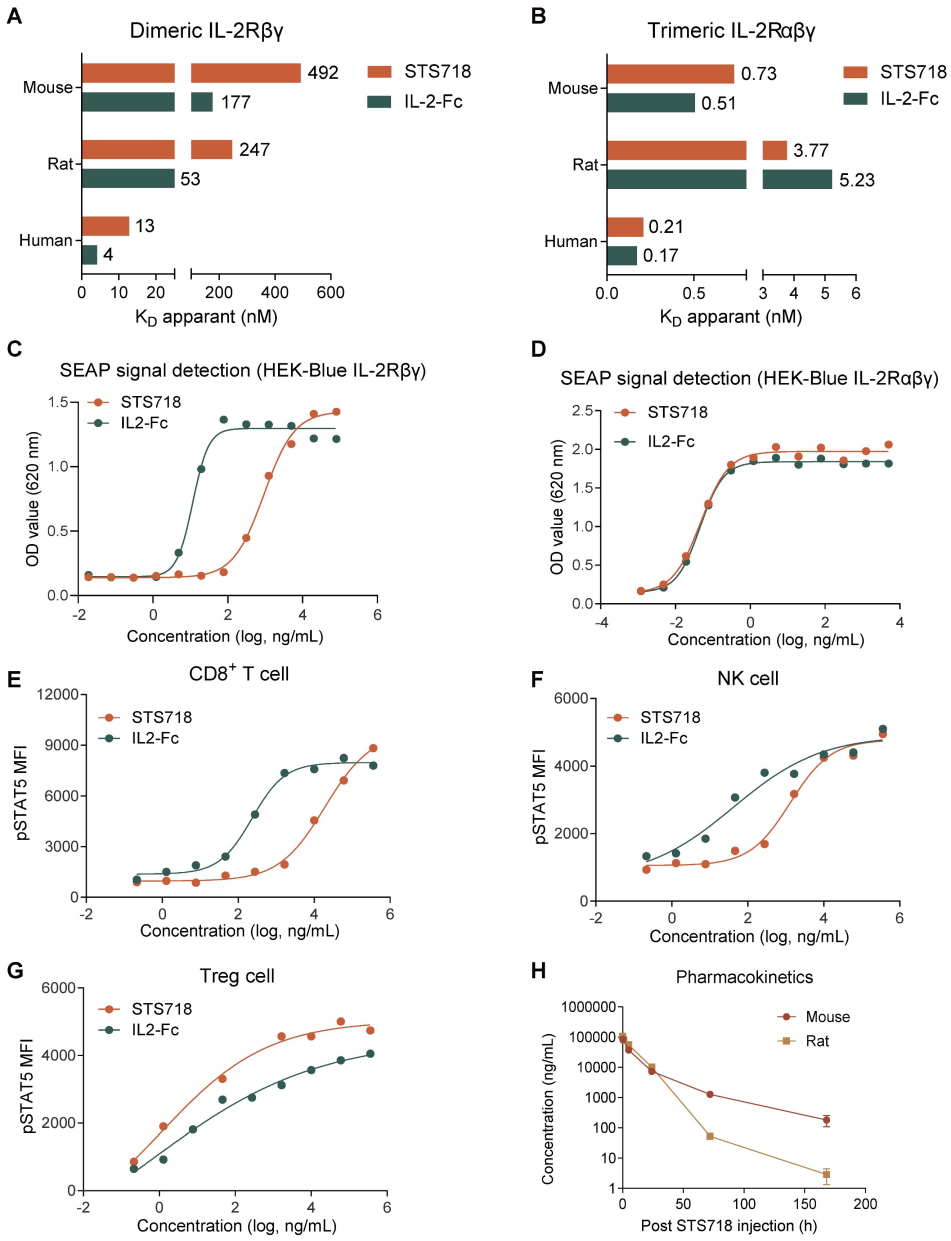
** Binding affinity for IL-2 receptor, *in vitro* bioactivity, and pharmacokinetics of STS718.** (**A**) Binding affinity (K_D_ apparent) was analyzed by surface plasmon resonance. Compared with human wild-type IL-2-Fc, STS718 has reduced affinity for the dimeric IL-2Rβγ across multiple species, including human, rat, and mouse. (**B**) The binding affinity for trimeric IL-2Rαβγ was comparable between STS718 and human wild-type IL-2-Fc. (**C-D**) HEK-Blue reporter cells expressing IL-2Rβγ or IL-2Rαβγ initiate the JAK3/STAT5 signaling and secrete SEAP enzyme upon IL-2 stimulation. Cells were treated with STS718 or wild-type IL-2-Fc. The IL-2 bioactivity was expressed as OD value with secreted SEAP signal indicating STAT5 phosphorylation. (**E-G**) The ability of STS718 and wild-type IL-2-Fc to induce STAT5 phosphorylation in human peripheral blood mononuclear cells. The phosphorylated STAT5 (pSTAT5) median fluorescence intensity (MFI) was analyzed on CD8^+^ T cells, NK cells, and CD4^+^CD25^+^Foxp3^+^ Treg cells by flow cytometry. For *in vitro* experiments, three technical replicates were averaged in each experiment; Data are expressed as mean only in (**A-G**). (**H**) Curves presented the changes of plasma concentrations of STS718 following a single intravenous injection of 1 mg/kg to C57BL/6J mice (*n* = 10) and Sprague-Dawley (SD) rats (*n* = 6). Blood samples were collected at different time points to determine the bioavailability of STS718 by ELISA. Data are expressed as mean ± SEM in (**H**).

The* in vitro* binding affinity of STS718 to IL-2Rβγ and IL-2Rαβγ across species, including human, rat, and mouse, was initially evaluated by SPR. For IL-2Rβγ, STS718 had 3-4 fold lower affinity (K_D_) compared to wild-type IL-2-Fc of human, rat, and mouse (**Figure 2A**). For IL-2Rαβγ, comparable high-affinity binding was observed between STS718 and wild-type IL-2-Fc across species (**Figure 2B**).

We next assessed the bioactivity of STS718 in HEK-Blue reporter cells and human PBMCs. HEK-Blue reporter cells were designed to activate the Janus activating kinase 3 (JAK3) / STAT5 pathway upon IL-2 stimulation, which constitutes the core pathway predominantly dominating the IL-2 signaling 30 (**Figure 1A**). STS718 or wild-type IL-2-Fc increased SEAP activity representing their bioactivity: the EC_50_ values with STS718 were far higher than that with wild-type IL-2-Fc in HEK-Blue CD122/CD132 cells (924.60 ng/mL versus 11.59 ng/mL, **Figure 2C**), while remained similar in HEK-Blue IL-2 cells (0.047 ng/mL versus 0.046 ng/mL, **Figure 2D**). When titrated on isolated human PBMCs, STS718 showed substantially reduced pSTAT5 activity (EC_50_ = 19169.00 ng/mL versus EC_50_ = 231.80 ng/mL) on CD8^+^ T cells and NK cells (EC_50_ = 1312.00 ng/mL versus EC_50_ = 41.39 ng/mL) compared to wild-type IL-2-Fc, while pSTAT5 activities were observed to be comparable in Treg cells (EC_50_ = 15.38 ng/mL versus EC_50_ = 4.52 ng/mL) (**Figure 2E-G**). Therefore, STS718 showed much lower bioactivity for IL-2Rβγ on effector cells, while maintaining comparable bioactivity to IL-2Rαβγ expressed on Treg cells *in vitro* compare to wild-type IL-2-Fc.

The pharmacokinetic characteristics of STS718 were assessed by a single intravenous dose of STS718 in C57BL/6J mice and SD rats. Wild-type human IL-2 has a plasma half-life (*T_1/2_*) of less than 10 min for intravenous administration 29. In contrast, whereas STS718 demonstrated markedly improved pharmacokinetics, with extended half-lives of approximately 23 h in mice and 9 h in rats (**Figure 2H**), through fusion with Fc regions which enhanced its stability* in vivo*.

### STS718 selectively expands Treg cells *in vivo* in mice and nonhuman primates

**Figure 3:**
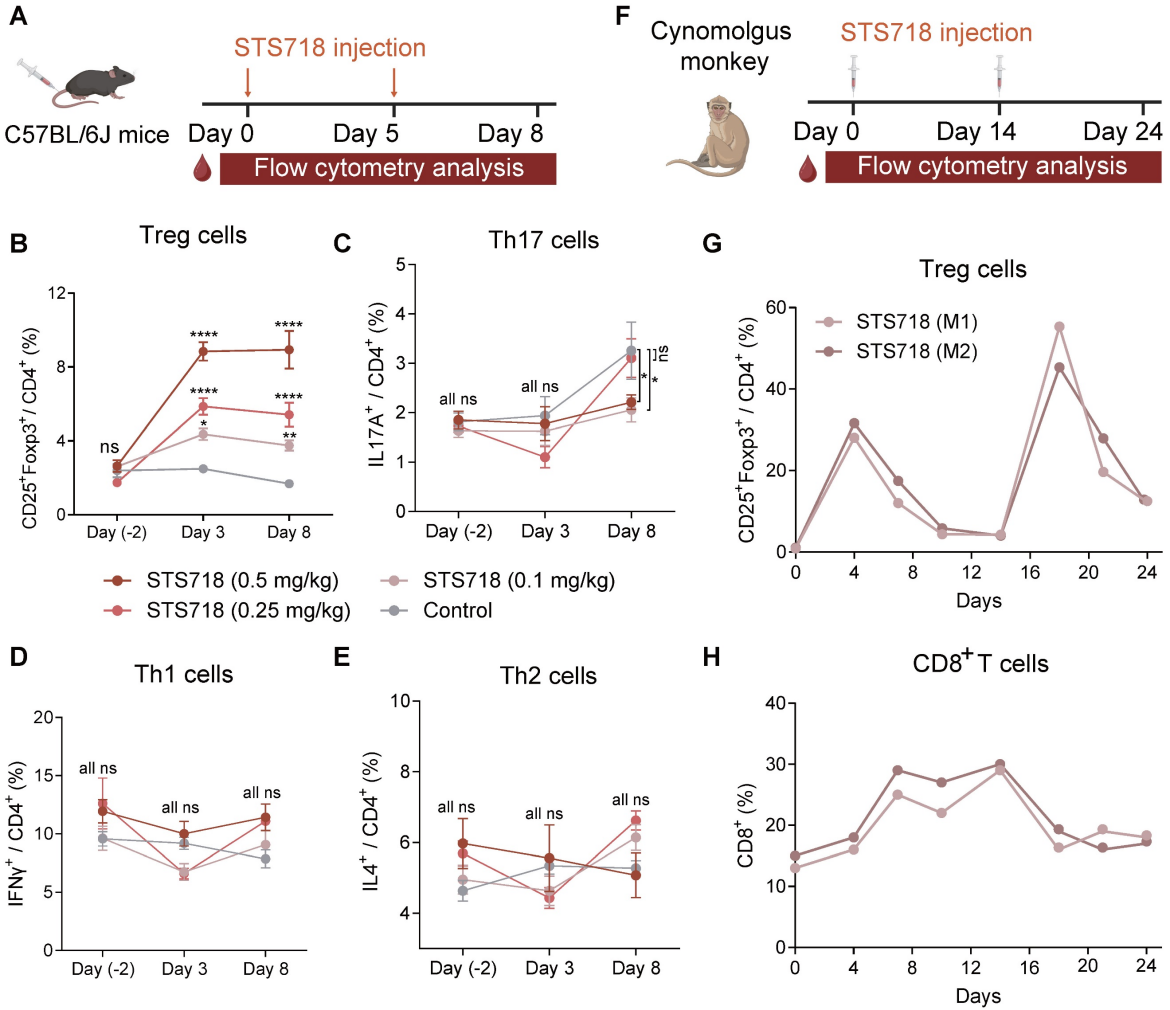
** STS718 selectively *in vivo* expands Treg cells in mice and cynomolgus monkeys.** (**A**) Schematic strategy to evaluate Treg selectivity of STS718 in C57BL/6J mice (*n* = 5 per group). Mice received a single intravenous administration of STS718 (0.1, 0.25, and 0.5 mg/kg) or control PBS on days 0 and 5. Percentages of CD25^+^Foxp3^+^ Treg cells (**B**), IL17A^+^ Th17 cells (**C**), IFNγ^+^ Th1 cells (**D**), and IL-4^+^ Th2 cells (**E**) in CD4^+^ T cell population on days -2, 3, and 8 were analyzed by flow cytometry. Data are expressed as mean ± SEM; statistical significance was determined by Two-way ANOVA test with Dunnett's multiple comparisons test. (**F**) Schematic strategy to evaluate Treg selectivity of STS718 in cynomolgus monkeys (*n* = 2). Cynomolgus monkeys received first subcutaneous administration of 0.3 mg/kg STS718 on day 0 and a second 1.0 mg/kg dosing on day 14. Percentages of CD4^+^CD25^+^Foxp3^+^ Treg cells (**G**) and CD8^+^ T cells (**H**) were analyzed by flow cytometry.

To assess the *in vivo* capacity of STS718 to expand Treg cells, C57BL/6J mice received intravenous injections at doses of 0.1, 0.25, or 0.5 mg/kg STS718 on day 0 and day 5 (**Figure 3A**). The expansion of circulating Treg cells reached its maximum on day 3, and maintained a stable elevation through day 8 in a dose-dependent manner, and mice receiving 0.5 mg/kg STS718 exhibited an approximately 3.5-fold expansion compared to PBS-treated controls (**Figure 3B**). After the second dosing shot, Treg cells could still hold a high percentage after three days. During the differentiation of CD4^+^ T cells, IL-2 regulates cytokine receptor and transcription factor expression, thereby suppressing T helper 17 cells (Th17) differentiation and promote the fate decisions of Th1 and Th2 cells 31. We evaluated the influence of STS718 administration on these effector T cell subtypes in mice. The proportion of circulating Th17 cells was markedly decreased on day 8 in mice receiving 0.1 and 0.3 mg/kg STS718, compared to controls (*P* < 0.05; **Figure 3C**). No time- or dose-dependent changes were observed in the proportion of circulating Th1 and Th2 cells (*P* > 0.05; **Figure 3D**-**E**).

To evaluate its *in vivo* functions in cynomolgus monkeys, STS718 was given subcutaneously at 0.3 mg/kg on day 0, and 1.0 mg/kg on day 14 (**Figure 3F**). The percentage of Treg cells in peripheral CD4^+^ T cells showed about 30-fold increase on day 4 following the first dose of 0.3 mg/kg STS718 compared with baseline (approximately 1% Treg cells gated in CD4^+^ T cells), and remained above the baseline until at least day 14. Following a second dose of 1.0 mg/kg STS718 on day 14, the percentage of Treg cells increased more substantially, peaking at approximately 50% of circulating CD4^+^ T cells on day 18 (**Figure 3G**). IL-2 administration typically promotes the expansion of CD8^+^ T cells, an effector population that constitutively express dimeric IL-2Rβγ 32. An approximately 2-fold peak expansion was noted in CD8^+^ T cells after the first dose of STS718, which was much lower than the expansion of Treg cells. Treg cells possess the inhibitory potential on effector cell expansion 18. In line with this theory, CD8^+^ T cells started to decline from day 14 even after a second STS718 dosing, in parallel with the increase of Treg cells (**Figure 3H**). Therefore, these data demonstrate that STS718 is capable of selectively expanding functional Treg cells *in vivo* in both mice and cynomolgus monkeys.

### STS718 treatment suppresses experimental autoimmune GN progression

To evaluate the potential utility of STS718 characterized by increased Treg function for treatment of autoimmune GN, we set up two rat models of experimental autoimmune GN with STS718 administration, including experimental CrN and experimental PHN. The two models represent human anti-GBM disease (the most aggressive type of autoimmune GN with rapid clinical course) 3 and membranous GN (the most frequent cause of adult nephrotic syndrome) 33, respectively.

**Figure 4:**
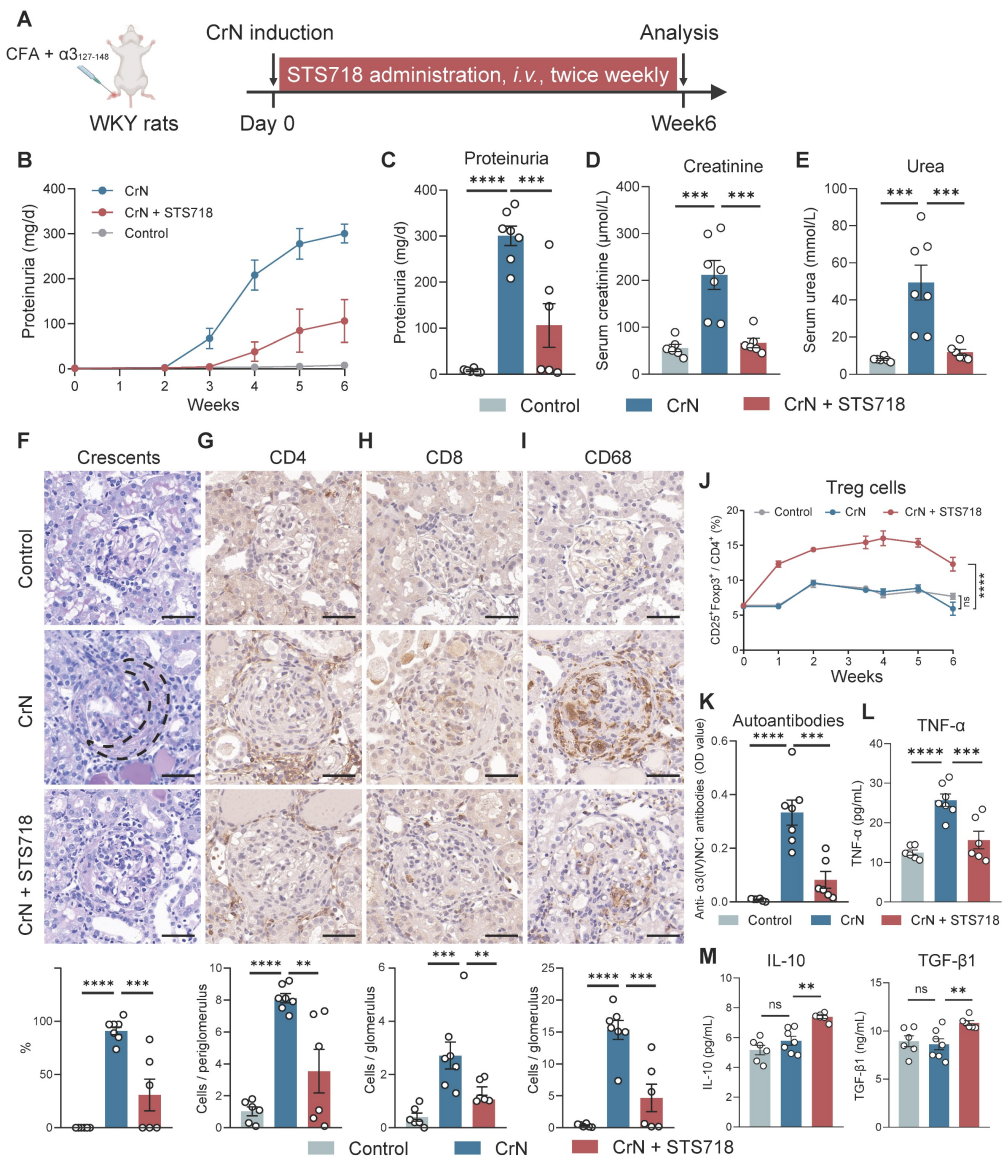
** STS718 suppresses the disease progression in a rat crescentic glomerulonephritis model.** (**A**) Schematic strategy to induce experimental crescentic glomerulonephritis (CrN) model. After immunization, Wistar-Kyoto (WKY) rats (*n* = 6-7 per group) were treated with intravenous administration of 0.3 mg/kg STS718 from day 0 to week 6. 24-h proteinuria (**B-C**), serum creatinine (**D**), and serum urea (**E**) were measured. (**F**) Representative Periodic Acid-Schiff staining of glomeruli lesions and quantitative analysis of crescents. Crescent was outlined in the vehicle-treated CrN group. (**G-I**) Representative immunohistochemistry staining of CD4^+^ T cells, CD8^+^ T cells, and CD68^+^ macrophages in the kidneys and corresponding quantitative analysis results. Scale bars = 50 μm. (**J**) Percentages of circulating CD4^+^CD25^+^Foxp3^+^ Treg cells during disease course. (**K**) Circulating autoantibodies against full-length human α3(IV)NC1 were measured. (**L-M**) Circulating levels of TNF-α, IL-10, and TGF-β1 were quantified. Data are expressed as mean ± SEM; statistical significance in (**C-I**,** K-M**) was determined by One-way ANOVA test with Dunnett's multiple comparisons test; statistical significance in (**J**) was determined by Two-way ANOVA test with Dunnett's multiple comparisons test.

Experimental CrN was established in WKY rats by active immunization with pathogenic α3_127-148_ peptide (**Figure 4A**). Peptide-immunized rats treated with vehicle alone for 3 weeks developed a severe CrN that shared features with human anti-GBM disease, including increased proteinuria (**Figure 4B-C**), high levels of serum creatinine and serum urea (**Figure 4D**-**E**), and diffuse glomerular crescent formation on kidney pathology (**Figure 4F**). In contrast, STS718 treatment significantly alleviated worsening kidney function and severe crescent formation (**Figure 4B-F**). Remarkable inflammation infiltration within glomerulus or peri-glomerulus was observed in CrN rats, including CD4^+^ T cells, CD8^+^ T cells, and CD68^+^ macrophages, while these inflammatory cells significantly decreased in CrN rats receiving STS718 treatment (**Figure 4G-I**). A significantly higher percentage of circulating Treg cells could be detected sustainably in STS718-treated CrN rats compared with these vehicle-treated rats, and remained a high level during the whole experimental period (**Figure 4J**). Autoantibodies against intact antigen α3(IV)NC1 were detected during course in α3_127-148_-immunized rats compared to healthy controls, while their levels dropped in STS718-treated rats (**Figure 4K**). Circulating levels of the pro-inflammatory cytokine tumor necrosis factor α (TNF-α) were increased in these vehicle-treated rats, while STS718 administration showed a decrease (**Figure 4L**). IL-10 and TGF-β1 are well-known effector factors for Treg cells to exert suppressive properties 18. We found that circulating levels of both IL-10 and TGF-β1 were elevated in STS718-treated rats (**Figure 4M**). Since STS718 is a humanized IL-2 mutein, we evaluated its immunogenicity *in vivo* by detecting antibodies against itself. Although circulating anti-STS718 antibodies appeared from week 2, they remained at a very low level, and showed a downward trend (**Figure S1**).

**Figure 5:**
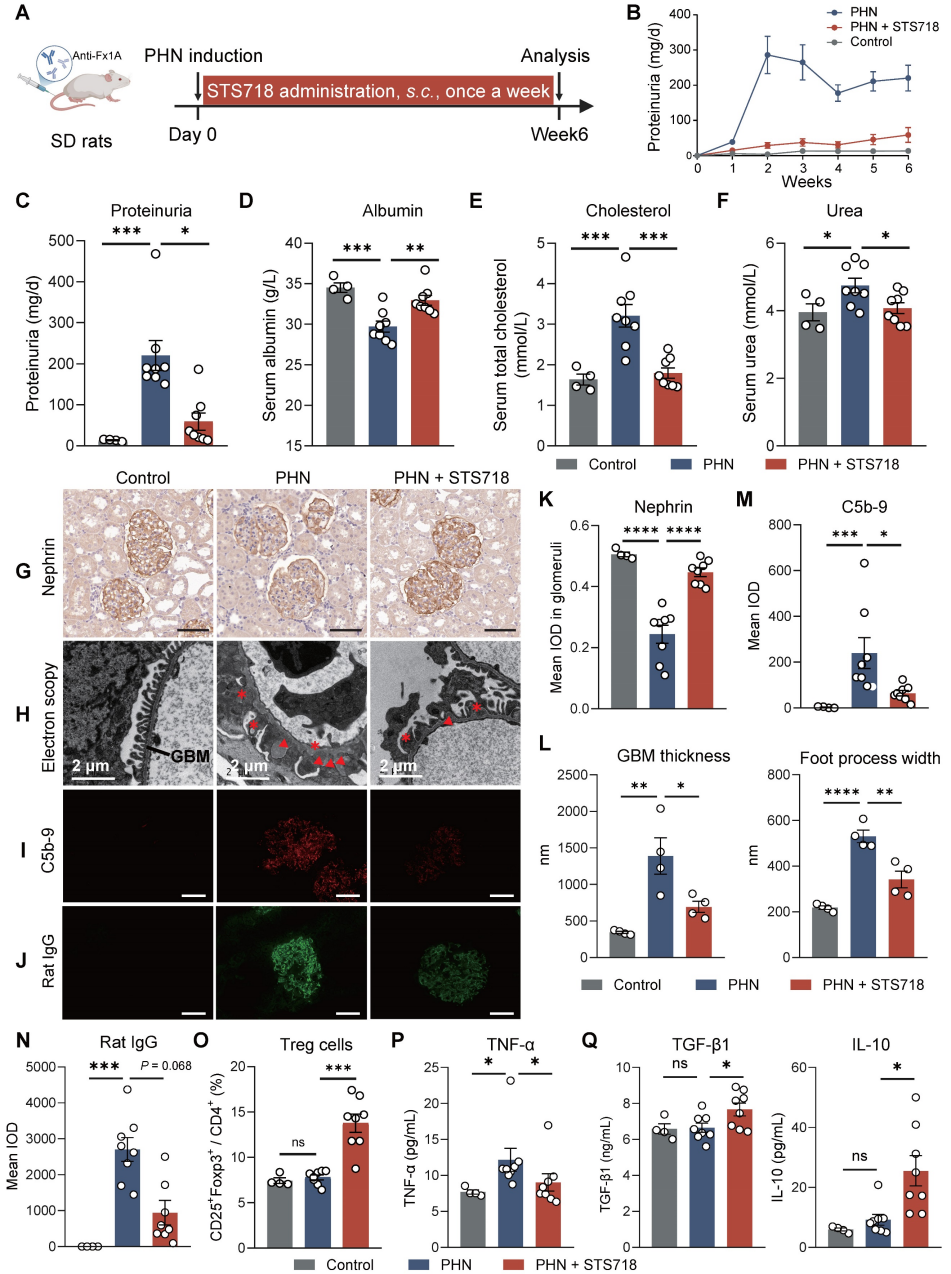
** STS718 reduces the disease severity in a rat membranous glomerulonephritis model.** (**A**) Schematic strategy to induce passive Heymann nephritis (PHN). After immunization, Sprague-Dawley (SD) rats (*n* = 4-8 per group) were treated with subcutaneous dose of 0.3 mg/kg STS718 from day 0 to week 6. 24-h proteinuria (**B-C**), serum albumin (**D**), serum total cholesterol (**E**), and serum urea (**F**) were measured. (**G**) Representative immunohistochemistry staining of podocyte nephrin and quantification results were shown in (**K**). Scale bars = 50 μm. (**H**) Representative electron microscopy observation of changes in glomerular filtration barrier morphology. Triangle indicates immune deposits and asterisk indicates effaced foot processes. Scale bars = 2 μm. The glomerular basement membrane (GBM) thickness and podocyte foot process width were quantified (*n* = 4 per group) and results were shown in (**L**). (**I-J**) Representative immunofluorescence staining of complement C5b-9 and rat IgG deposits within glomerulus and corresponding quantification results were shown in (**M-N**). Scale bars = 50 μm. (**O**) Percentages of circulating CD4^+^CD25^+^Foxp3^+^ Treg cells. (**P-Q**) Circulating levels of TNF-α, IL-10, and TGF-β1 were quantified. Data are expressed as mean ± SEM; statistical significance in (**D-F, K-L, O**) and in (**Q**) with analysis of TGF-β1 was determined by One-way ANOVA test with Dunnett's multiple comparisons test; statistical significance in (**C, M-N, P**) and in (**Q**) with analysis of IL-10 was determined by Kruskal-Wallis's test with Dunn's multiple comparisons test.

We further assessed the efficacy of STS718 in the PHN model. Because SD rat weight increases at a higher speed than that of WKY rat, we conducted subcutaneous STS718 administration in this project, a route that also offers practical utility for clinical management. PHN was built via passive immunization of sheep anti-rat Fx1A antibodies (**Figure 5A**). The phenotype was observed quickly over one week, included massive proteinuria (**Figure 5B**-**C**), hypoalbuminemia (**Figure 5D**), hyper-cholesterolemia (**Figure 5E**), and a mild kidney function injury (**Figure 5F**). When treated with STS718 once a week, these symptoms were significantly alleviated correspondingly (**Figure 5B-F**). Podocyte injury and complement activation were dominant features in PHN rats, resembling human membranous GN. In detail, STS718 administration could mitigate podocyte nephrin loss (core cytoskeleton component), foot process effacement, and GBM thickening, and reduced complement C5b-9 deposits (**Figure 5G-I and Figure 5K-M**). Immunofluorescence staining also showed decreased glomerular rat IgG deposits in STS718-treated rats compared with PHN rats (**Figure 5J and 5N**), and the glomerular sheep IgG deposits were comparable between the two groups (**Figure S2A-B**). The expansion of Treg cells was also noted after STS718 administration (**Figure 5O**). STS718 administration resulted in decreased circulating levels of TNF-α as well as increased levels of IL-10 and TGF-β1 (**Figure 5P-Q**).

To assess the therapeutic potential of STS718 following disease establishment, we administered STS718 to PHN rats from week 3 onwards, once severe proteinuria had developed (**Figure S3A**). STS718 treatment showed a trend in reducing the levels of proteinuria compared with vehicle-treated group, though statistically significance did not reach (**Figure S3B-C**). However, the loss of serum albumin and levels of serum creatinine were decreased in STS718-treated rats (**Figure S3C**). Additionally, STS718 still could markedly increase proportions of Treg cells in both the blood and spleen (**Figure S3D-G**).

Taken together, these *in vivo* findings demonstrate that IL-2 mutein STS718 treatment could efficiently suppress autoimmunity-derived inflammation, showing therapeutic potential in a broad spectrum of autoimmune kidney diseases.

### STS718 exhibits superior therapeutic potential to wild-type IL-2 in experimental autoimmune GN

**Figure 6:**
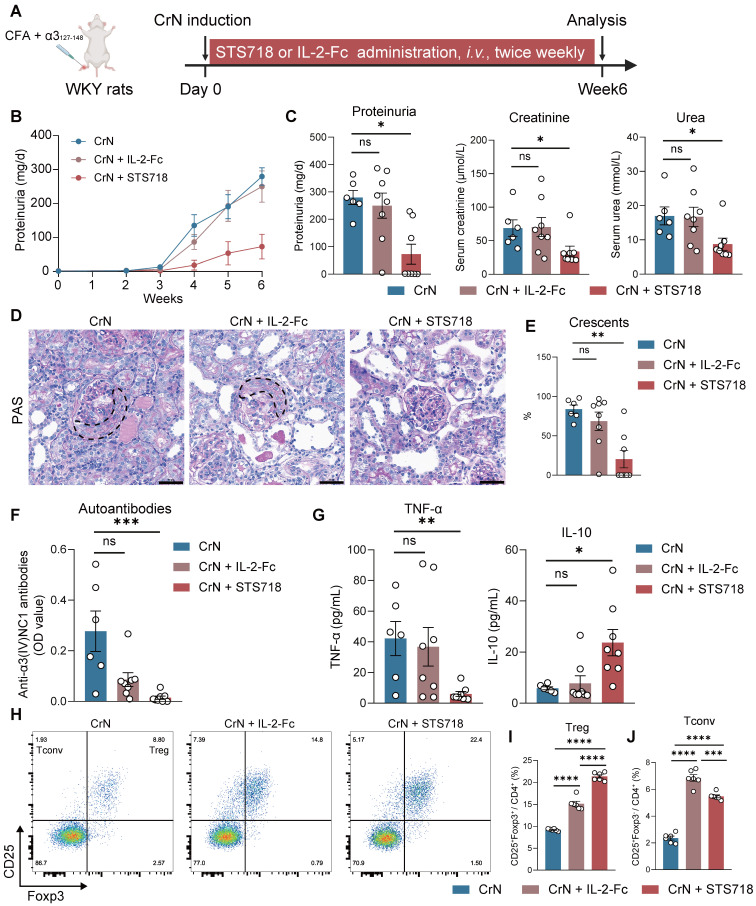
** Therapeutic comparison of STS718 and wild-type IL-2-Fc in rats with crescentic glomerulonephritis.** (**A**) Schematic strategy to induce experimental crescentic glomerulonephritis (CrN) model. After immunization, Wistar-Kyoto (WKY) rats (*n* = 6-8 per group) were treated with intravenous administration of 0.3 mg/kg STS718 or wild-type IL-2-Fc from day 0 to week 6. (**B-C**) 24-h proteinuria, serum creatinine, and serum urea were measured. (**D-E**) Representative Periodic Acid-Schiff (PAS) staining of glomeruli lesions and quantitative analysis of crescents. Crescents were outlined. Scale bars = 50 μm. (**F**) Circulating autoantibodies against full-length human α3(IV)NC1 were measured. (**G**) Circulating levels of TNF-α and IL-10 were measured. (**H**) Representative flow cytometry analyses of circulating CD4^+^CD25^+^Foxp3^+^ Treg cells and CD4^+^CD25^+^Foxp3^-^ Tconv cells (*n* = 6 per group), and quantified results were shown in (**I-J**). Data are expressed as mean ± SEM; statistical significance in (**C, E, F, G**) was determined by Kruskal-Wallis test with Dunn's multiple comparisons test; statistical significance in (**I, J**) was determined by One-way ANOVA test with Tukey's multiple comparisons test.

**Figure 7:**
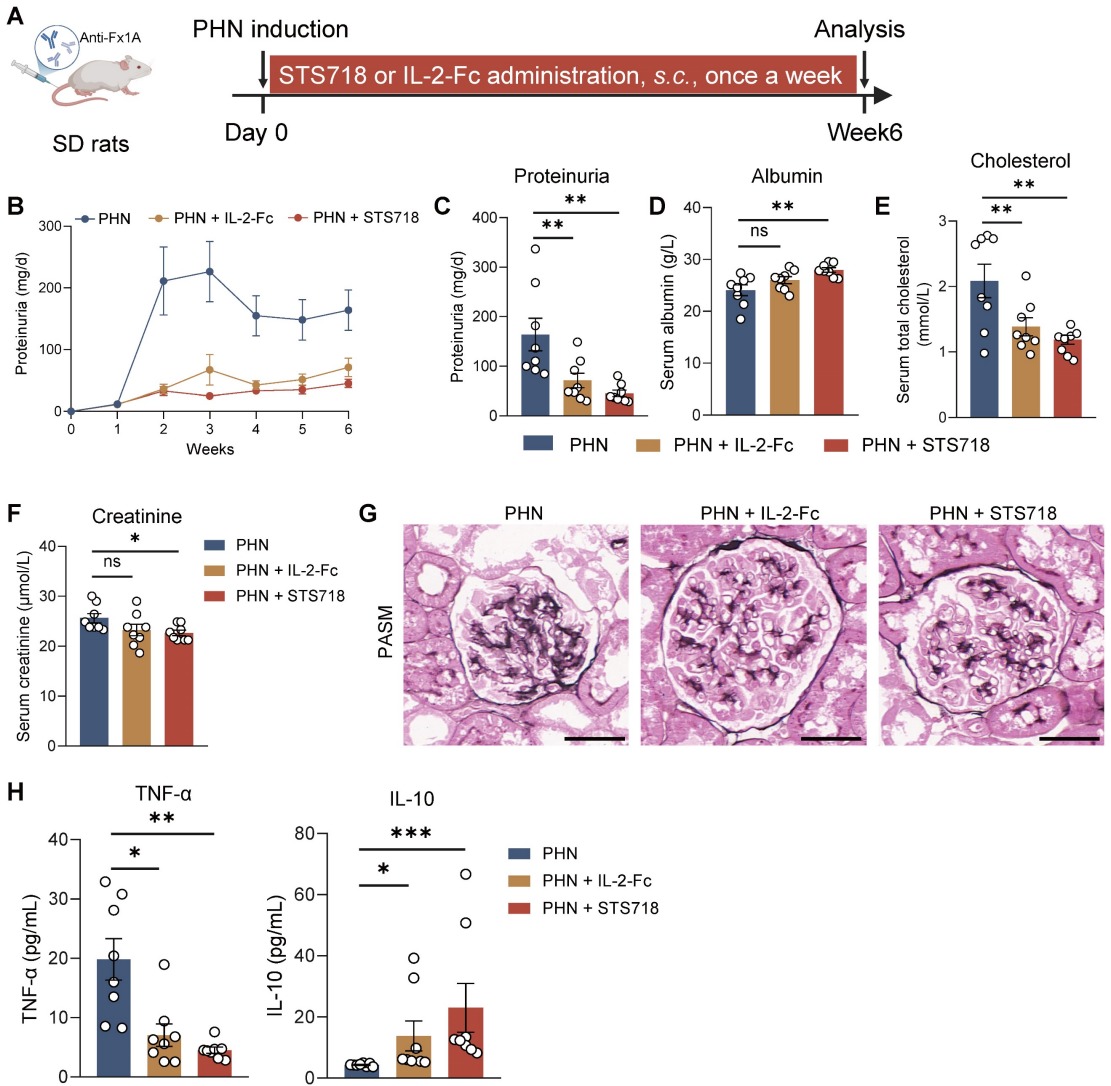
** Therapeutic comparison of STS718 and wild-type IL-2-Fc in rats with membranous glomerulonephritis. (A)** Schematic strategy to induce passive Heymann nephritis (PHN). After immunization, Sprague-Dawley (SD) rats (*n* = 8 per group) were treated with subcutaneous dose of 0.3 mg/kg STS718 or wild-type IL-2-Fc from day 0 to week 6. (**B-F**) 24-h proteinuria, serum albumin, serum total cholesterol, and serum creatinine were measured. (**G**) Representative Periodic-acid silver methenamine (PASM) staining showing the glomerular basement membrane thickening. Scale bars = 50 μm. (**H**) Circulating levels of TNF-α and IL-10 were measured. Scale bars = 50 μm. Data are expressed as mean ± SEM; statistical significance in (**C-E**) was determined by One-way ANOVA test with Dunnett's multiple comparisons test; statistical significance in (**F, H**) was determined by Kruskal-Wallis's test with Dunn's multiple comparisons test.

To further assess the efficacy of STS718, we next compared equivalent doses of wild-type IL-2-Fc and STS718 in the two aforementioned rat models of experimental autoimmune GN. In experimental CrN, treatment with wild-type IL-2-Fc developed similar level of disease severity to that observed in vehicle-treated rats, characterized by proteinuria, impaired kidney function, and diffuse crescent formation (**Figure 6A-E**). However, consistent with previous findings, STS718 administration markedly reduced proteinuria and mitigated kidney function injury and severe crescent formation (**Figure 6A-E**). Compared with vehicle-treated rats, STS718-treated rats showed decreased levels of circulating autoantibodies and TNF-α, as well as increased IL-10. However, these changes were not observed in rats with wild-type IL-2-Fc treatment (**Figure 6F-G**). Although equal doses were used, STS718 treatment resulted in a higher frequency of circulating CD25^+^Foxp3^+^ Treg cells but a lower frequency of CD25^+^Foxp3^-^ conventional T cells (Tconv cells) than with wild-type IL-2-Fc (**Figure 6H-J**). When evaluating the efficacy of wild-type IL-2-Fc and STS718 in PHN, both treatments showed effects in lowering massive proteinuria (**Figure 7A-C**), levels of serum cholesterol (**Figure 7E**) and mitigating kidney pathology (**Figure 7G**). However, STS718 demonstrated superior features in alleviating loss of serum albumin (**Figure 7D**) and reducing inflammatory responses, as evidenced by lower levels of serum creatinine (**Figure 7F**) and TNF-α, while exhibiting increased IL-10 levels, compared with wild-type IL-2-Fc (**Figure 7H**). Collectively, these data provide further support for the superior efficacy and targeted Treg selectivity of this engineered IL-2 mutein STS718 in treating autoimmune GN.

### STS718 enhances the differentiation of Treg cells in healthy donors and autoimmune GN patients

**Figure 8:**
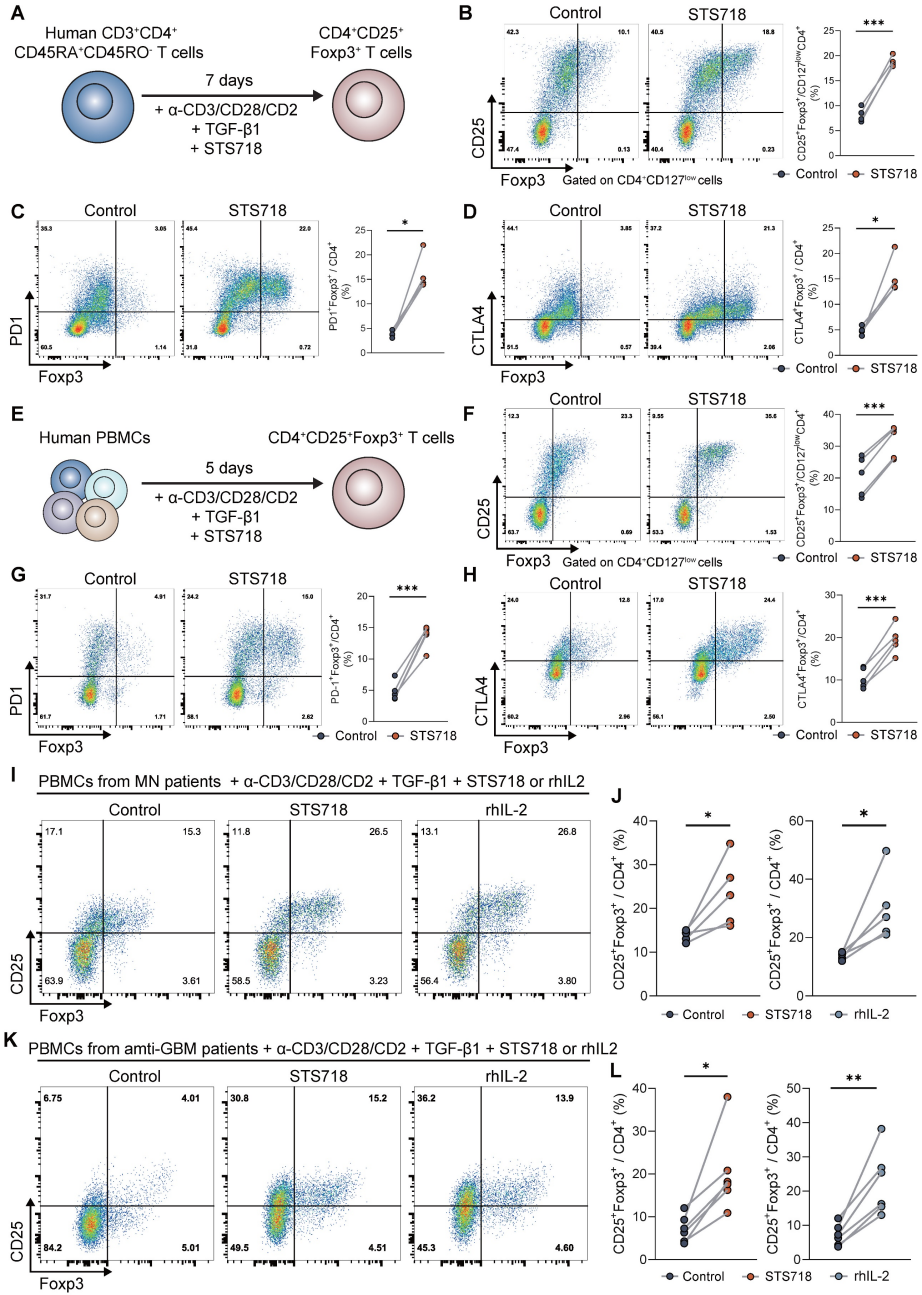
** STS718 expands human Treg cells from healthy donors and autoimmune glomerulonephritis patients.** (**A**) Schematic strategy to test the ability of STS718 to expand Treg cells in naïve CD4^+^ T cell of healthy donors (*n* = 4) under Treg differentiation conditions. (**B-D**) Representative flow cytometry analyses of CD4^+^CD127^low^CD25^+^Foxp3^+^, CD4^+^PD1^+^Foxp3^+^, and CD4^+^CTLA4^+^Foxp3^+^ expressions in naïve T cells given PBS or STS718 (100 ng/mL) and corresponding line graph. (**E**) Schematic strategy to test the ability of STS718 to expand Treg cells in peripheral blood mononuclear cells (PBMCs) of healthy donors (*n* = 5) under Treg differentiation conditions. (**F-H**) Representative flow cytometry analyses of CD4^+^CD127^low^CD25^+^Foxp3^+^, CD4^+^PD1^+^Foxp3^+^, and CD4^+^CTLA4^+^Foxp3^+^ expressions in naïve T cells given PBS or STS718 (100 ng/mL) and corresponding line graph. (**I-L**) PBMCs from autoimmune glomerulonephritis patients, including membranous glomerulonephritis/nephropathy (MN,* n* = 5) and antiglomerular basement membrane (anti-GBM) disease (*n* = 6) were cultured under Treg differentiation conditions in the presence of STS718 (100 ng/mL) or recombinant human IL-2 (rhIL-2, 10 ng/mL). Treg cells were analyzed by flow cytometry; **I**) Representative flow cytometry analyses and (**J**) line graphs of CD4^+^CD25^+^Foxp3^+^ expression in MN patients; **K**) Representative flow cytometry analyses and (**L**) line graphs of CD4^+^CD25^+^Foxp3^+^ expression in anti-GBM patients. Statistical significance was determined by paired t test. Three technical replicates were averaged in each experiment.

We further sought to confirm whether STS718 could promote the expansion of Foxp3^+^ Treg cells in human T cells. Naïve CD4^+^ T cells of healthy donors were isolated and activated by anti-CD3/CD28/CD2 antibodies and TGF-β1, to evaluate the effects of STS718 (**Figure 8A**). CD4^+^CD127^low^CD25^+^Foxp3^+^, CD4^+^PD1^+^Foxp3^+^, and CD4^+^CTLA4^+^Foxp3^+^ T cell populations were significantly increased after 7-days culture with STS718 (**Figure 8B-D and Figure S4**). These Treg subpopulations were also similarly elevated in the settings of PBMCs from healthy donors upon STS718 stimulation (**Figure 8E-H**). We finally investigated whether STS718 could also induce Foxp3 expression in the context of PBMCs of autoimmune GN patients, including membranous GN and anti-GBM disease. PBMCs of patients exposed to STS718 exhibited a significant rise in CD4^+^CD25^+^Foxp3^+^ Treg cells after activation (**Figure 6I-L**). Because no previous studies have reported the rhIL-2 therapeutic potential in both membranous GN and anti-GBM disease, we here also tested its effect on PBMCs of these patients and found a comparable ability like STS718 to induce Treg expansion (**Figure 6I-L**). Collectively, these results demonstrated that Treg induction via rhIL-2 or its mutein STS718 is feasible for autoimmune GN patients.

## Discussion

In the present study, we showed that an engineered IL-2 mutein STS718 had a low affinity to dimeric IL-2Rβγ across species, including human, rat, and mouse compared with wild-type IL-2-Fc. STS718 exhibited an extended serum half-life compared to rhIL-2, due to fusion with Fc regions. These unique properties enable STS718 to preferentially amplify Treg cells *in vivo* within a well-tolerated dosing window without apparently stimulating effector T cells. We demonstrated the therapeutic potential of STS718, administered either subcutaneously or intravenously, to be greater than that of wild-type IL-2-Fc in experimental autoimmune GN. Finally, we confirmed that STS718 could induce expansion of Treg cells from naïve T cells of healthy donors and PBMCs of autoimmune GN patients. These findings offer proof-of-concept support for IL-2 mutein-based immunotherapy to minimize unspecific immunosuppression and represent an alternative choice for effective treatment of autoimmune GN.

*In vivo* expansion of Treg cells via low-dose IL-2 represents an emerging and promising therapeutic therapy for autoimmune diseases, including systemic lupus erythematosus 34, rheumatoid arthritis 35, and primary Sjögren Syndrome 36. However, although these studies are encouraging, the narrow therapeutic window and considerable heterogeneity of IL-2 administration render it hard to establish a “universal” dosage with selectively robust induction of Treg cells in all patients 37, 38. The frequencies of dimeric IL-2Rβγ pathogenic effector T cells among diverse autoimmune diseases are varied, promoting the need for personalizing IL-2 dose to achieve optimal Treg selectivity. In addition, the brief half-life of IL-2 determines its frequent dosing in clinical settings. These limitations spurred the development for novel IL-2-based biologics exhibiting robust Treg partiality and enhanced pharmacokinetics.

The resultant STS718 was selected from a YSD library of IL-2 muteins through a strategy of reducing the binding affinity for IL-2Rβγ while maintaining comparable affinity for IL-2Rα relative to wild-type IL-2. Sequencing analysis revealed that STS718 had undergone a mutation with 81RPRDL85 was replaced with RHL. This mutation resulted in the diminished hydrogen bond interaction between IL-2 and IL-2Rβ. Under physiological conditions, the decreased affinity between IL-2 and IL-2Rβ makes IL-2 more prone to bind IL-2Rα-expressing cells, such as Treg cells 39. Several IL-2 muteins were developed to enhance Treg selectivity by introducing point mutations at their interface with IL-2Rβ, such N88D 40 or H16L 39 in human IL-2 or N103R and V106D in murine IL-2 13. These mutations, together with our current mutations in STS718, provide key insights for deciphering the complexity of IL-2 biology. The shifted decreased affinity of STS718 to IL-2Rβγ was further confirmed across species by SPR, and validated by pSTAT5 assays using human HEK-reporter cells as well as PBMCs, indicating a successful engineering for Treg selectivity. Besides, the short pharmacological half-life of IL-2 was overcome in the design of STS718 through fusion to a human IgG1 Fc domain.

With the reduced binding affinity *in vitro*, we further assessed the capacity of STS718 to promote Treg expansion *in vivo*. Circulating Treg cells in mice administered STS718 increased significantly over time in a dose-dependent manner, while the percentage of effector Th1 and Th2 cells remained inactive. We confirmed that STS718 was more potent in cynomolgus monkeys for Treg induction than that in mice, with approximately 30-fold expansion in Treg cells compared with baseline and remained elevated last for 14 days after dosing. Treg cells have inhibitory function on effector T cells through multiple mechanisms, such as secreting regulating anti-inflammatory regulatory mediators (e.g., IL-10, IL-35, and TGF-β1) and soaking up extracellular IL-2 that attenuating pro-inflammatory signaling 18. Although a relatively mild increase (~ 2-fold) in CD8^+^ T cell population was observed after the first STS718 administration to cynomolgus monkeys, this population decreased after the second STS718 dosing, while the expansion of Treg cells population increased substantially again. These findings may indirectly indicate that STS718 could induce functional Treg cells in nonhuman primates.

Although engineered IL-2 muteins have been applied in cancer 32, transplantation 39, and autoimmune rheumatic diseases 41, studies on their applications in autoimmune GN are scarce. Our current study expanded this therapeutic strategy to a broad spectrum of autoimmune GN by showing the efficacy of STS718-induced expansion of Treg cells in different models of experimental autoimmune GN. Autoimmune GN patients comprise a substantial population suffering from a spectrum of kidney diseases characterized by the autoimmunity directed against the kidneys 1. Treg-mediated immune tolerance is elucidated elegantly in autoimmune GN 42. Impaired IL-2 function has been associated with human membranous GN, suggesting the potential benefits of IL-2 based therapies 43. Besides, reduced percentage of Treg cells at active stage in peripheral blood 44, 45 and corresponding elevations in response to anti-CD20 immunotherapy 46 were observed in membranous GN patients. These clues imply that Treg-based therapies may provide potential benefits in controlling progression of autoimmune GN patients. In the current study, the designed STS718 showed superior features to the wild-type IL-2-Fc that directly *in vivo* expanded a number of Treg cells to control progression of rat models of autoimmune GN. Moreover, treatment of PBMCs of autoimmune GN patients with STS718 or just rhIL-2 resulted in an increase of Treg cells. Taken together, these findings represent a potential translational insight for the future utility of IL-2-based therapeutics in autoimmune GN patients.

Limitations are acknowledged in our study. First, due to topic-centered, the efficacy of STS718 was only tested in experimental autoimmune GN models, including crescentic GN and membranous GN. Given that STS718 has the ability to induce Treg cells across species, it is reasonable to speculate that STS718 might be a valuable experimental tool for studying mechanisms or therapeutic concepts in various preclinical models beyond autoimmune GN. Second, we have only examined the effect of STS718 on total CD4^+^ T subtype cells, CD8^+^ T cells, and NK cells. Other subsets of immune cells that express dimeric IL-2Rβγ, such as effector memory CD4^+^ and CD8^+^ T cells, are also needed to be further explored.

## Conclusion

In conclusion, we engineered an IL-2 mutein, STS718, with diminished binding affinity for IL-2Rβγ, enabling high-effective, thereby enable potent and selective expansion of Treg cells* in vivo*. Proof-of-concept studies demonstrated that STS718 treatment suppressed the progression of experimental autoimmune GN and induce Foxp3^+^ Treg cells in PBMCs of autoimmune GN patients. These findings provide a novel immunotherapy strategy for autoimmune GN, with the potential to minimize nonspecific immunosuppression.

## Supplementary Material

Supplementary methods, figures and tables.

## Data Availability

All data that support the findings are included within the paper. Other source data related to this study are available from the corresponding authors upon reasonable request.

## Abbreviations

Treg: regulatory T
Tconv: conventional T
IL-2: interleukin-2
IL-2R: interleukin-2 receptor
rhIL-2: recombinant human interleukin-2
GN: glomerulonephritis
Foxp3: transcription factor forkhead box protein 3
FACS: fluorescence-activated cell sorting
SPR: surface plasmon resonance
SEAP: secreted embryonic alkaline phosphatase
EC_50_: median effective concentration
MFI: mean fluorescence intensity
pSTAT5: phosphorylated signal transducer and activator of transcription 5
CrN: crescentic glomerulonephritis
PHN: passive Heymann nephritis
CFA: complete Freund's adjuvant
TNF-α: tumor necrosis factor α
TGF-β1: transforming growth factor β1
Th1: T helper 1
Th2: T helper 2
Th17: T helper 17
PBMCs: peripheral blood mononuclear cells
PD-1: programmed death 1 receptor
CTLA-4: cytotoxic T lymphocyte-associated antigen-4
MN: membrane nephropathy
GBM: glomerular basement membrane
