# Supplementary material for: In vivo Expansion of Regulatory T cells via an Engineered IL-2 Mutein to Suppress Autoimmune Glomerulonephritis
Source: Theranostics. 2026 Feb 18;16(9):4787–803. doi: 10.7150/thno.126588 (PMC12964244; doi:10.7150/thno.126588)
Supplement: Supplementary file 1 — Supplementary methods, figures and tables. [file thnov16p4787s1.pdf]

Supplemental file for

***In vivo* expansion of regulatory T cells via an engineered IL-2 mutein to suppress autoimmune glomerulonephritis**

Huang Kuang, et al.

***Correspondence to: constancej@163.com; cuizhao@bjmu.edu.cn**

This PDF file includes:

Supplemental methods

Figure S1

Figure S2

Figure S3

Figure S4

Table S1

Table S2

Table S3

Supplemental methods for animal experiments

Assessment of disease severity.

24-h urine and blood specimens were sampled prior to and following immunization, with weekly sampling intervals. Proteinuria and serum creatinine, urea, albumin, and total cholesterol were quantified using an automated biochemical analyzer (UniCel DxC 600 Synchron; Beckman Coulter, Inc.). Kidney tissues were evaluated using light microscopy, immunofluorescence, and electron microscopy. For light microscopic analysis, kidney tissues were fixed in 10% neutral buffered formalin, paraffin-embedded, and sectioned (3 µm thick). Tissue sections were subjected to staining with periodic acid–Schiff (PAS) or Periodic-acid silver methenamine (PASM), and a minimum of 100 glomeruli per section were examined to assess the crescents lesion. For immunofluorescence analysis, frozen kidney sections (5 µm thick) were acetone-fixed and incubated with anti-C5b-9 antibodies (sc-66190; Santa Cruz) overnight at 4°C, following incubation of goat anti-mouse IgG H&L (AF555) highly cross-adsorbed secondary antibody (A32727; Invitrogen). Anti-Rat IgG (FITC) (F6258; Sigma-aldrich) and anti-Sheep IgG H&L (AF555) (A-21436; Invitrogen) antibodies were used for detecting the deposition of rat and sheep IgG. The mean immunofluorescence intensity of C5b-9 deposits was quantified by Image-Pro Plus (v6.0; Media Cybernetics). For electron microscopic analysis, samples were sequentially fixed in 3% glutaraldehyde and 1% osmium tetroxide, dehydrated through a graded ethanol series, and embedded in Epon 812 resin. Ultrathin sections were contrasted with uranyl acetate and lead citrate before examination under a transmission electron microscope (JEM-1230; JEOL).

To assess podocyte foot process effacement, two glomeruli were randomly chosen from specimens. For each glomerulus, 15–20 non-overlapping digital images were acquired at 13,000× magnification. The number of foot processes per micrometer of glomerular basement membrane (GBM) and GBM thickness were calculated using ImageJ (v1.48, NIH, Bethesda, MD). The FPW was calculated using the arithmetic mean formula:

$$PFW = \frac{4}{\pi} \times (\frac{\sum GBM\ length}{\sum foot\ process\ count})$$

A correction factor of π/4 was applied to account for random foot process orientation in ultrathin sections.

Immunohistochemistry

Kidney sections were dehydrated and subjected to antigen retrieval using one of the following methods: (1) heat-induced epitope retrieval in citric acid buffer (pH 6.0) for CD4 and CD8 detection, or EDTA buffer (pH 9.0) for nephrin staining, both performed under high-temperature and high-pressure conditions for 4 minutes; or (2) enzymatic antigen retrieval using 0.04% pepsin at 37°C for 30 minutes for CD68 detection. After blocking endogenous peroxidase with 3% H_2O_2 and nonspecific binding with 3% BSA, the sections were incubated overnight at 4°C with the following antibodies: anti-CD4 (25229S; Cell Signaling Technology), anti-CD8 (sc-53063; Santa Cruz), anti-CD68 (MCA341GA; Bio-Rad), or anti-nephrin (ab216341; Abcam) antibodies. Staining was further visualized using 3,3′-diaminobenzidine (DAB). For quantitative analysis, a minimum of 10 random glomeruli per sample were evaluated. $CD4^+$ T cells, $CD8^+$ T cells, and $CD68^+$ macrophages were quantified as the number of positive cells per glomerular or peri-glomerular cross-section. Nephrin expression was measured as mean integrated optical density (IOD) per glomerular cross-section by Image-Pro Plus.

Circulating antibody and cytokines detection by enzyme-linked immunosorbent assay

Antibodies towards full-length α3(IV)NC1 and STS718 in rat experimental crescentic glomerulonephritis model were analyzed by ELISA. α3(IV)NC1 (2 μg/ml, home-made) and STS718 (2 μg/ml) were coated overnight on the 96-well plates. Plasma samples were diluted 1:100 in PBS containing 0.1% Tween-20, and incubated in plates for 1 hour at 37°C. After washing, alkaline phosphatase (AP)-conjugated rabbit anti-rat IgG (A6066, Sigma-Aldrich), diluted 1:5000 in PBS with 0.1% Tween-20, was added and incubated for 45 minutes at 37°C. The reaction was developed using p-nitrophenyl phosphate in substrate buffer, and absorbance was measured spectrophotometrically at 405 nm. The levels of circulating cytokines were detected by commercial ELISA kits

following the manufacturer's protocols: Rat TNF-α ELISA kit (E-HSEL-R0001, Elabscience), Rat IL-10 ELISA kit (E-HSEL-R0005, Elabscience), Rat TGF-β1 ELISA kit (CSB-E04727r, CUSABIO)

Detection of Treg cells by flow cytometry

The proportion of circulating Treg cells in rat autoimmune glomerulonephritis models was detected by flow cytometry. 40 μL whole blood was acquired from rats and stained with antibodies against CD4 and CD25 for 20 minutes. Following red blood cell lysing (00-4300-54; Invitrogen) and washing, cells were then fixed and permeabilized using Foxp3/Transcription Factor Staining Buffer Set (00-5523-00; Invitrogen) for 45 minutes, and stained with antibodies against Foxp3 for 30 minutes. Cells were analyzed using FACS Verse (BD Biosciences) and FlowJo software v10.8. The proportions of Treg cells and Tconv cells were presented as $CD25^+Foxp3^+$ expression and $CD25^+Foxp3^-$ in gated $CD4^+$ T cells, respectively.

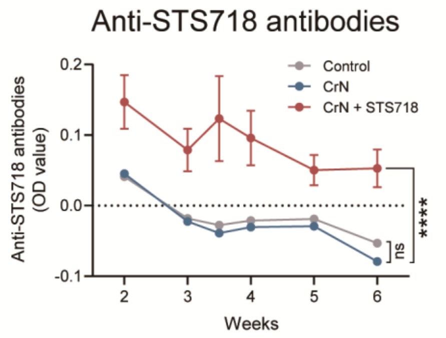

Figure S1. Circulating antibodies against STS718 were measured. Data are expressed as mean ± SEM; statistical significance in was determined by Two-way ANOVA test with Dunnett's multiple comparisons test.

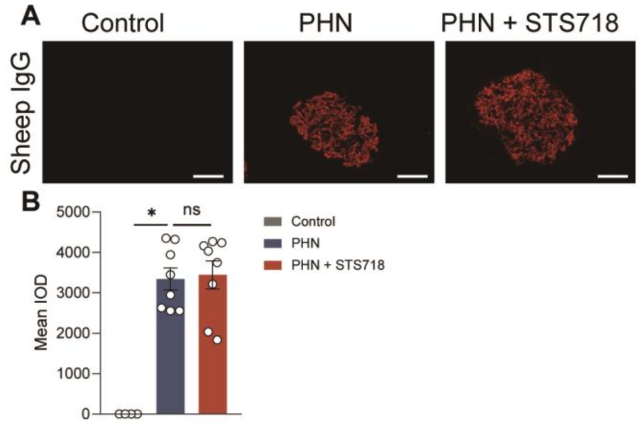

Figure S2. Glomerular sheep IgG deposits in passive Heymann nephritis (PHN) was detected. (**A**) Representative immunofluorescence staining of sheep IgG deposits within glomerulus. (**B**) Shown is quantification of glomerular sheep IgG deposits. Data are expressed as mean ± SEM; statistical significance was determined by Kruskal-Wallis's test with Dunn's multiple comparisons test.

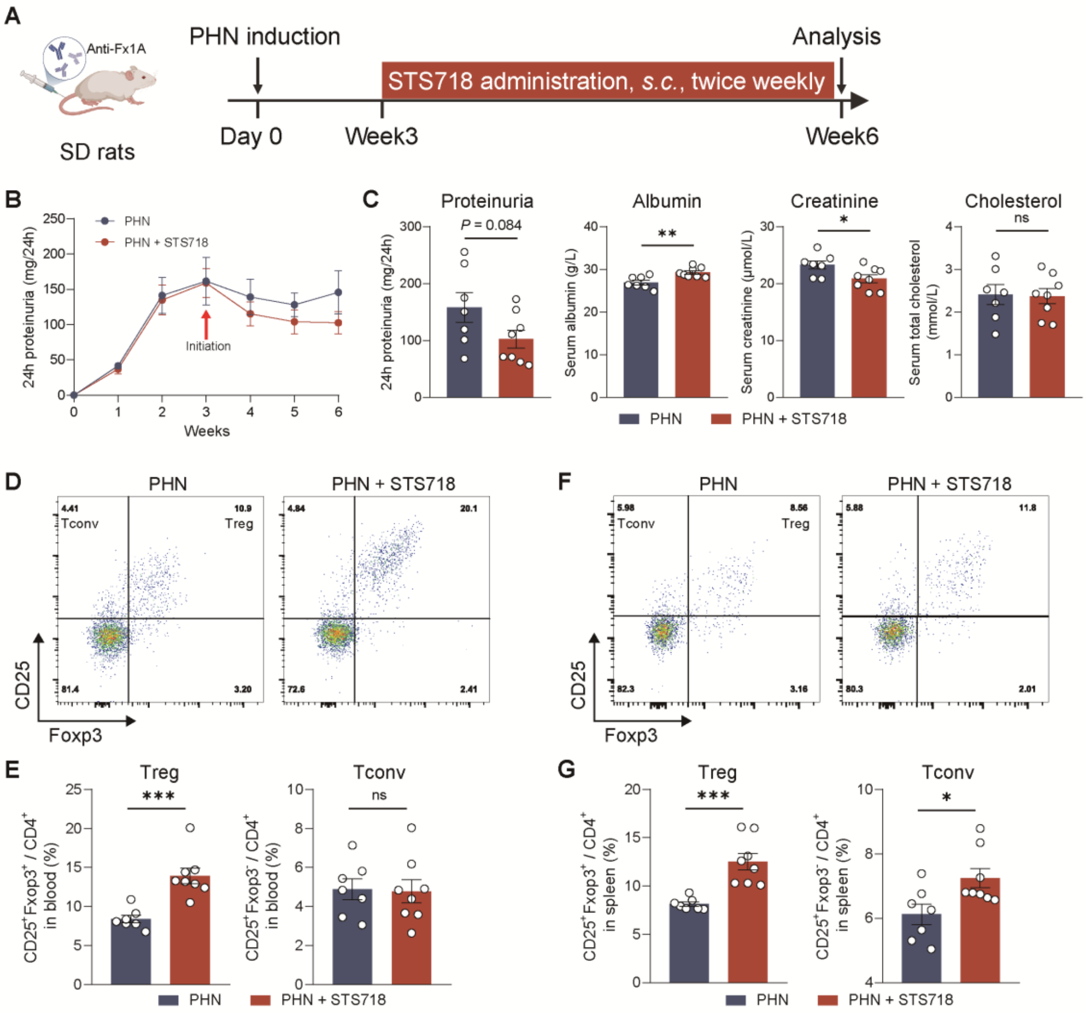

Figure S3. Therapeutic intervention with STS718 in rats with established membranous glomerulonephritis. (**A**) Schematic strategy to induce passive Heymann nephritis (PHN). Three weeks after immunization, Sprague-Dawley (SD) rats (*n* = 7~8 per group) were treated with subcutaneous dose of 0.3 mg/kg STS718 twice weekly. (**B-C**) 24-hour proteinuria, serum albumin, serum total cholesterol, and serum creatinine were measured. (**D, F**) Representative flow cytometry analyses of $CD4^{+}CD25^{+}Foxp3^{+}$ Treg cells and $CD4^{+}CD25^{+}Foxp3^{-}$ Tconv cells from blood and spleen, and quantified results were shown in (**E, G**). Data are expressed as mean ± SEM; statistical significance was determined by unpaired t test.

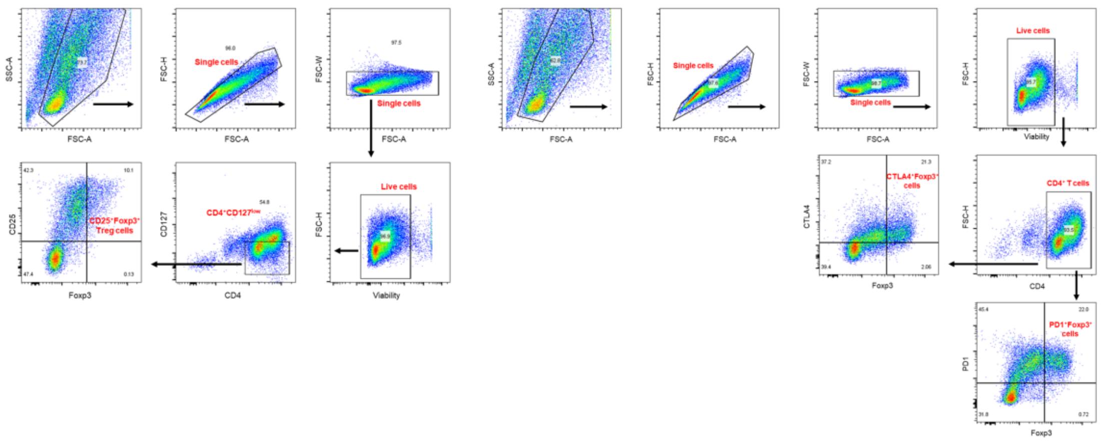

Figure S4. Gating strategy for cell subsets in human naïve T cells upon STS718 stimulation.

Supplemental Table 1. Information for antibodies or relevant agents in flow cytometry.

Antibodies or relevant agents	Source	STS718 experiments
Streptavidin-PE	554061, BD Biosciences	Screening
THE™ V5 Tag Antibody [iFluor 647]	A01805-100, GenScript	
CD3-BV421	E-AB-F1230Q2, Elabscience	Bioactivity in human PBMCs (panel 1)
CD8-PE	AN00427D, Elabscience	
CD56-AF488	E-AB-F1270L, Elabscience	
pSTAT5-AF647	562076, BD Biosciences	
CD4-BV421	562424, BD Biosciences	Bioactivity in human PBMCs (panel 2)
CD25-AF647	356128, Biolegend	
pSTAT5-PE	612567, BD Biosciences	
Foxp3-AF488	320112, Biolegend	
CD4-FITC	100406, Biolegend	Treg selectivity in mice
CD25-APC	102012, Biolegend	
Foxp3-PE	320008, Biolegend	
IL4-APC	504106, Biolegend	
IFNγ-BV421	505829, Biolegend	
IL17A-PE	506904, Biolegend	
CD4-Pacific Blue™	317429, Biolegend	Treg selectivity in cynomolgus monkeys
CD25-AF647	356128, Biolegend	
Foxp3-AF488	320112, Biolegend	
CD8-BV605™	344742, Biolegend	
CD4-FITC	E-AB-F1352C, Elabscience	Treg expansion in human naïve T cells and PBMCs of healthy donors (panel 1)
CD127-Percp/Cy5.5	E-AB-F1152J, Elabscience	
CD25-PE-Cy7	E-AB-F1194H, Elabscience	
Foxp3-PE	320008, Biolegend	
CD4-FITC	E-AB-F1352C, Elabscience	Treg expansion in human naïve T cells and PBMCs of healthy donors (panel 2)
PD1-PE-Cy7	561272, BD Biosciences	
CTLA4-APC	369612, Biolegend	
Foxp3-PE	320008, Biolegend	
CD4-APC	E-AB-F1109E, Elabscience	Treg expansion in human PBMCs of patients
CD25-FITC	E-AB-F1194C, Elabscience	

Foxp3-PE	320008, Biolegend	
CD4-PerCP/Cyanine5.5	201520, Biolegend	Treg detection in rat models
CD25-PE	12-0390-82, Invitrogen	
Foxp3-AF647	320014, Biolegend	
Zombie NIR™ Fixable Viability Kit	423106, Biolegend	Cell viability staining
Fixation Buffer	554655, BD Biosciences	*In vitro* pSTAT5 assays
Phosflow™ Perm Buffer III	558050, BD Biosciences	
Foxp3/Transcription Factor Staining Buffer Set	00-5523-00, Invitrogen	Detection for Treg cells
Cell Activation Cocktail	423304, Biolegend	Detection of Th1/Th2/Th17 cells
Cyto-Fast™ Fix/Perm Buffer Set	426803, Biolegend	

Supplemental Table 2. Demographic information of enrolled patients with membranous nephropathy.

	MN1	MN2	MN3	MN4	MN5
Gender	Male	Female	Male	Male	Male
Age, year	60	32	47	42	67
Serum creatinine, µmol/L	372.8	236.6	237.7	85	91
Serum albumin, g/L	16.5	37.9	17.5	25.4	29.5
Proteinuria, g/day	13.78	2.81	20.47	5.89	2.4
Anti-PLA2R1 antibody, RU/mL	101.57	191	1078	229.9	1500

MN, membranous nephropathy; PLA2R1, phospholipase A2 receptor 1.

Supplemental Table 3. Demographic information of enrolled patients with anti-GBM disease.

	GBM1	GBM2	GBM3	GBM4	GBM5	GBM6
Gender	Female	Female	Female	Female	Female	Female
Age, year	70	35	37	46	51	28
Serum creatinine, µmol/L	450.9	84.92	333.7	275	357.9	318.7
eGFR, mL/min per 1.73 m^2	7.98	76.806	14.479	17.174	12.143	16.306
Serum urea, mmol/L	25.66	8.26	15.55	10.06	23.8	19.65
Anti-GBM antibody, RU/mL	162	127	112	26	87	180
Crescents on kidney biospy, %	76.5	40	62.2	52.2	71.4	71.4

eGFR, estimated glomerular filtration rate; GBM, glomerular basement membrane.
